# Infra-Low Frequency Neurofeedback in the Treatment of Patients With Chronic Eating Disorder and Comorbid Post-Traumatic Stress Disorder

**DOI:** 10.3389/fnhum.2022.890682

**Published:** 2022-05-06

**Authors:** Anna Winkeler, Markus Winkeler, Hartmut Imgart

**Affiliations:** Department of Psychosomatics and Psychotherapy, Parkland-Klinik, Bad Wildungen, Germany

**Keywords:** neurofeedback, infra-low frequency, eating disorder, post-traumatic stress disorder, randomized control trial, symptom reduction

## Abstract

The treatment of patients suffering from an eating disorder and a comorbid post-traumatic stress disorder is challenging and often leads to poor outcomes. In a randomized control trial, we evaluated to what extent adding Infra-Low Frequency (ILF) neurofeedback could improve symptom reduction within an established inpatient treatment program. In a randomized two-group design, patients suffering from an eating disorder (anorexia nervosa, bulimia nervosa, or binge eating disorder) and comorbid post-traumatic stress disorder (*N* = 36) were examined while attending an inpatient treatment program in a clinic for psychosomatic disorders. The intervention group received ILF neurofeedback in addition to regular therapy, while the control group received “media-supported relaxation” as a placebo intervention. At the beginning and at the end of their treatment, all participants completed the Eating Disorder Examination-Questionnaire (EDE-Q) as a measure of eating disorder psychopathology and the Impact of Event Scale-Revised (IES-R) in order to assess symptoms of post-traumatic stress. Changes in EDE-Q and IES-R scores over time served as primary outcomes as well as an increase in body mass index in underweight patients. Secondary outcomes were the perceived benefit of the received intervention, global assessment of psychological treatment success, and complications in the course of treatment. Statistical evaluation was carried out with repeated measurement analysis of variance for the primary outcomes and with *t*-tests and Fisher’s exact test for the secondary outcomes. Our results indicate better treatment outcomes in the ILF neurofeedback group with regard to trauma-associated avoidance as well as with regard to restraint eating and increase in body weight. Furthermore, patients who had received ILF neurofeedback rated the intervention they received and, in tendency, their overall treatment more positively and they experienced fewer complications in the course of treatment. ILF neurofeedback is very well accepted by patients and seems to provide a relevant additional benefit in some aspects of symptom reduction. Findings confirm the feasibility of embedding this treatment approach in an inpatient setting and support the case for a larger study for greater statistical power.

**Clinical Trial Registration**: “Infra-Low Frequency Neurofeedback training in the treatment of patients with eating disorder and comorbid post-traumatic stress disorder”; German Clinical Trials Registry (https://www.drks.de; Identifier: DRKS00027826).

## Introduction

Eating disorders (ED) cause severe suffering and are associated with massive impairment and a reduced life expectancy. Mortality rates are more than five times higher for anorexia nervosa (AN) and 1.5 times higher for bulimia nervosa (BN) and binge eating disorder (BED) than would be expected for the respective age group in the general population (Fichter and Quadflieg, 2016). According to DSM-5 (American Psychiatric Association, 2013), ED in females has a 12-month prevalence between 0.4% (AN) and 1.6% (BED).

Anorexia nervosa (ICD-10: F50.0; World Health Organization, 2004) is characterized by self-induced weight loss or persistent effort to stay underweight through dietary restriction and/or measures to counteract weight gain, such as vomiting or excessive exercise. Despite being underweight, those affected often perceive themselves as fat and have a pathological fear of gaining weight. Patients with BN (ICD-10: F50.2) also suffer from preoccupation with food and body shape, and recurrent binge eating with loss of control, followed by vomiting, use of laxatives, or other forms of counter-regulation. BED, which is coded in ICD-10 under F50.8 (“other eating disorders”) and only has an independent diagnosis in DSM-5, is also characterized by recurrent binge eating with loss of control but differs from BN with regard to the absence of measures to counteract weight gain and is therefore often associated with obesity. The treatment results for ED, especially AN and BN, are not satisfactory. In their review of the epidemiology, course, and outcome of eating disorders, Smink et al. (2013) found a 5-year recovery rate of 69% for AN and 55% for BN, whereas Steinhausen (2002) reported lifetime remission rates of just under 50% for AN in adults.

Between 9% and 24% of patients with ED suffer from comorbid post-traumatic stress disorder (PTSD; Rijkers et al., 2019). PTSD (ICD-10: F43.1) can occur after one or more events of exceptional threat, such as rape, physical violence, or natural disasters. It is characterized by reliving the traumatic event in the form of intrusive trauma-associated memories, flashbacks, or nightmares, as well as avoidance behaviors regarding trauma-associated experiences, circumstances, and locations. In addition, those affected suffer from persistent psycho-vegetative hyperarousal, which manifests itself, for example, in sleep disorders, inner tension, and restlessness as well as constant alertness. The course of PTSD is often chronic; in more than 30% of cases, complete remission cannot be achieved even with psychotherapy (Bradley et al., 2005).

As a new diagnosis, the ICD-11 includes complex PTSD as a symptom picture that is caused by particularly severe, long-lasting, and repetitive traumatic events such as sexual, physical, or emotional abuse or neglect during childhood (so-called type II traumata) and, in addition to the classic PTSD symptoms, can be characterized by dissociative episodes, self-harming behavior, self-loathing, and body disgust (Maercker et al., 2013). In addition to the core symptom domains of intrusive re-experiencing, avoidance of trauma reminders, and hyperarousal, the ICD-11 diagnosis of complex PTSD also requires the presence of symptoms from the three domains of disturbance of self-organization (i.e., difficulties in perceiving and regulating emotions and managing relationships and a fundamentally negative self-perception). For a detailed differential diagnostic description of complex PTSD, see Ford and Courtois (2021). A significant statistical connection between different forms of type II trauma and the occurrence of ED could be clearly shown in several reviews and meta-analyses (Caslini et al., 2016; Molendijk et al., 2017; Trottier and MacDonald, 2017). ED patients who have experienced childhood maltreatment are more likely to show psychiatric comorbidities and suicidal tendencies than patients without such a history and they suffer from a higher severity of the ED pathology with more frequent binge eating and purging behavior (Molendijk et al., 2017).

There is evidence that the association between PTSD following child abuse and ED symptom severity is mediated by physiological arousal and avoidance (Holzer et al., 2008), as well as dissociation and emotional dysregulation (Moulton et al., 2015). Rodríguez et al. (2005) found that women with ED who had been victims of sexual and other forms of violence had poorer ED treatment outcomes, were more likely to drop out of treatment, and had more relapses than women with ED who had not experienced violence. This is consistent with the authors’ clinical experience that the treatment of patients with ED and (complex) PTSD is usually more difficult and the ED symptoms improve less over time than in ED without serious comorbidities, even in an intensively supportive inpatient treatment setting. Many of our patients state that they use starvation and/or binge eating and vomiting to alleviate or temporarily end aversive trauma-associated emotional states such as shame, anger, and disgust, which makes it very difficult for them to refrain from ED symptoms. Thus, in accordance with Trottier and MacDonald (2017), ED in these patients can be understood as an attempt to cope with the consequences of their traumatization, such as hyperarousal symptoms, trauma-associated cognitions, and negative emotions. This maladaptive strategy is ultimately contributing to the perpetuation of both the ED and PTSD. In view of the unsatisfactory treatment results in a substantial proportion of cases in patients with ED and co-occurring PTSD, research into additional treatment options is clearly needed. One such option is Infra-Low Frequency neurofeedback.

Neurofeedback (NF) describes a specific form of biofeedback in which certain components of brain activity are measured, processed, and reported back to the patient in the form of visual, auditory, and/or tactile feedback. With electroencephalography (EEG)-NF, in which the feedback is generated from parts of the electrical activity on the surface of the skull, the goal is an optimized brain function that should manifest itself in improvements in well-being and coping with everyday life. NF has been increasingly used and researched in the treatment of a wide variety of disorders for years, such as attention deficit disorders (Van Doren et al., 2019) and addictive behavior (Sokhadze et al., 2008; Dousset et al., 2020).

In infra-low frequency neurofeedback (ILF NF), very slow electrical potential shifts (below 0.1 Hz), which are filtered out of the EEG signal, are used to generate the feedback. The continuous real-time feedback of brain activity in the ILF region initiates—unlike other types of NF with an operant conditioning model—an implicit learning process that targets the basic level of arousal and the extent of excitability of the central nervous system. For a more in-depth explanation of the process and the assumed mechanisms of action, see Othmer (2016). Dobrushina et al. (2020) were able to show that within a single ILF NF session significant modulation, measurable by fMRI, can be brought about in the intrinsic connectivity networks (ICN) of the brain. The ICN are of central importance for controlling the level of arousal in the organism and directing attention, also in response to external stimuli. These networks are disrupted in their functioning in PTSD patients including reduced connectivity of the default mode network in a resting state compared to healthy controls (Bluhm et al., 2009) and increased connectivity within the salience network, which may contribute to hypervigilance and hyperarousal symptoms in PTSD (Sripada et al., 2012; Lanius et al., 2015).

Reviews of the use of different forms of NF in patients with chronic PTSD including the period from 1991 to 2017 by Reiter et al. (2016) and Steingrimsson et al. (2020), as well as recent studies (e.g., van der Kolk et al., 2016) provide evidence for a clinical benefit of NF, which could in several studies also be linked to measurable neurophysiological changes such as altered connectivity of the default mode network and the salience network (Kluetsch et al., 2014; Nicholson et al., 2020). However, no RCTs on the use of ILF NF in PTSD are available to date, although some impressive case studies have been reported (e.g., Othmer and Legarda, 2011; Gerge, 2020). RCTs using ILF NF are also lacking in ED, although there are some encouraging results for other forms of NF on this subject, none however address the comorbidity of ED and PTSD. Lackner et al. (2016) were able to show significant positive effects of alpha frequency training on several characteristics of disturbed eating behavior and emotional skills in adolescent girls with AN, and recent RCTs have shown the effectiveness of different types of frequency band training and slow cortical potential training in women with subclinical and clinical BED in terms of reducing binge eating episodes (Schmidt and Martin, 2016; Blume et al., 2021).

In the light of the previous findings, which suggest that NF can be of clinical use in the treatment of both PTSD and ED, its application also appears promising in the difficult-to-treat group of patients who suffer from both disorders. The aim of this study was therefore to investigate the effectiveness of ILF NF in patients with chronic ED and comorbid PTSD. In order to check the feasibility and incremental benefit of ILF NF for this patient group in a realistic setting, ILF NF was embedded as an additional component in an existing inpatient treatment program. The patient group treated with ILF NF was compared with a placebo control group with regard to changes in ED symptoms, PTSD symptoms, BMI increase, perceived benefit of the intervention received, global subjective evaluation of the inpatient treatment, as well as the rate of complications during the course of treatment.

## Materials and Methods

### Study Design

A randomized control trial was conducted using a within-between-subjects design. Participants attending an inpatient treatment program designed for ED and PTSD were randomized either to an NF group or to a placebo control group and were assessed at the beginning of their inpatient treatment and after 12 sessions of either NF or placebo intervention.

The randomization was performed in a balanced manner by using the True Random Number Generator freely available online^1^ to generate six blocks of equal length permuted with regard to the two treatment groups, which were arranged in random order on the randomization list. The highest random numbers in each block were assigned to the intervention group, and the lowest to the placebo control group. According to the randomization list created in this way, the test subjects who met the inclusion criteria and had given their consent were assigned to the study groups.

The Consensus on the Reporting and Experimental Design of clinical and cognitive-behavioral Neurofeedback studies (CRED-nf; Ros et al., 2020) was followed as far as possible. Since the two study conditions obviously differed from each other, neither the experimenter nor the participants could be blinded during treatment.

In order to detect a medium-sized interaction effect of *f* = 0.25 in the analysis of variance (Cohen, 1988), which could be expected in light of previous studies with a similar target, a total sample size of *N* = 34 was necessary to realize an adequate statistical power of 1-ß = 0.80, a level of significance of *α* = 0.05, and two measurement times (Faul et al., 2007). Due to the expected drop-outs, *N* = 36 participants were to be recruited for the study.

### Participants

The study included patients aged 18 or over who underwent an inpatient treatment program for eating disorders at the Parkland-Klinik for psychosomatics and psychotherapy between May 2019 and April 2021. The presence of the relevant disorders was checked in a telephone interview with a clinical psychologist prior to inpatient admission. Inclusion criteria were the diagnosis of an ED according to ICD-10, i.e., AN (F50.0), atypical AN (F50.1), BN (F50.2), atypical BN (F50.3), BED (F50.8), and co-occurring diagnosis of PTSD (F43.1) or incomplete PTSD (F43.8). These diagnoses were established in a clinical interview by the attending therapist and validated by the head psychotherapist and the senior physician in a second interview. Exclusion criteria were treatment experience with NF as well as epileptic seizures in patients’ history. The reason for this was a certain risk that ILF NF might, especially in the initial phase of training, trigger paroxysmal symptoms, which usually disappear after individual adjustments to the training protocol (Othmer, 2017). While some of these symptoms, referred to in the theoretical framework of the ILF NF as instabilities of the nervous system, such as headaches or panic attacks, appeared tolerable to a certain extent, the occurrence of a more serious condition such as an epileptic seizure should not be risked in the context of the study.

### Ethical Approval

Written informed consent for participation was obtained from all participants before entering the study and after a detailed explanation of study procedures. The protocol followed the Declaration of Helsinki for the rights of the participants and the procedure of the study. The Ethical Review Board of the Landesärztekammer Hessen (regional medical association) approved the design and procedure of the study (Reference number FF 121/2018). Participants were not remunerated.

### Primary Outcomes

Primary outcome measures were facets of ED psychopathology and post-traumatic stress symptoms as well as Body Mass Index (BMI) assessed at the start of treatment and at the completion of intended treatment after 12 sessions.

For assessing the facets of symptomatology, participants completed the following self-report questionnaires:

Eating Disorder Examination-Questionnaire (EDE-Q; Hilbert and Tuschen-Caffier, 2006) as a measure of ED psychopathology with the four subscales *restraint* (i.e., restrained eating behavior), *eating concern*, *weight concern*, and *shape concern*, each of them ranging from 0 to 6. Participants were asked to answer the items with regard to the last 7 days.

Impact of Event Scale-Revised (IES-R; Maercker and Schützwohl, 1998) as a measure of post-traumatic stress with the three subscales *avoidance* (of cognitive or behavioral contact with the traumatic situation), *hyperarousal*, and *intrusion* (i.e., flashbacks, ruminations, and disturbing thoughts about the trauma). Participants were asked to focus on the most severe traumatic event in their lives. The test values ranged from 0 to 35 (subscales intrusion and hyperarousal) and from 0 to 40 (subscale avoidance), respectively.

For assessing BMI, patients underwent regular supervised weight controls. As a primary outcome, BMI was analyzed only for participants who were underweight before treatment (BMI <18.5 kg/m^2^).

### Secondary Outcomes

Patients’ *subjective benefit* of the received intervention (ILF NF vs. placebo control group, see below) was assessed on a scale ranging from 0 to 10 at the end of their treatment.

Patients’ *global assessment of psychological treatment success* (GAPS) was assessed on a scale constructed for the purpose of the study based on five items from the German basic documentation in psychotherapy (Heuft et al., 1998) being part of a more extensive questionnaire on satisfaction with the inpatient stay that is completed voluntarily by patients upon discharge. For these items, patients were asked to assess on a 5-point scale (1 = much improved, 2 = slightly improved, 3 = unchanged, 4 = slightly worsened, 5 = much worsened) to what extent their mental state, their self-esteem, their future orientation, their understanding of the disorder, and their general well-being have changed as a result of inpatient treatment. The GAPS scale showed a high internal consistency (Cronbach’s *α* = 0.85) with acceptable corrected item-total correlations (0.48 ≤*r_it_*≤ 0.79).

*Complications in the course of treatment* were recorded and classified based on the documentation in the medical record: non-compliance with weight agreement, severe self-injury, suicidal behavior.

### Treatments

#### Treatment Setup

Participants in both groups received 12 individual sessions lasting about 40 min, which included a short conversation about the course of the symptoms since the last session, followed by 30 min of ILF NF or placebo intervention. All sessions were conducted by staff trained in the procedures used and took place twice a week over 6 weeks in a quiet room. Participants were seated in a comfortable armchair placed in front of a monitor with speakers. After 30 min, the participants were asked to what extent their condition had changed during the session.

#### ILF Neurofeedback

The NF system by BEE Medic (BEE Medic GmbH, Singen, Germany), consisting of the NeuroAmp EEG Amplifier and the Cygnet biofeedback software was used for ILF NF. EEG signals were recorded from Ag/AgCl sintered electrodes positioned at the T4-P4 sites, T3-T4 sites, and in some of the patients also at the T4-Fp2 sites according to the international 10–20 system. The ground electrode was placed on the mastoid. The skin was prepared with NuPrep abrasive paste, and the electrodes were fixed with 10–20 conductive paste.

The sessions were conducted according to the protocol guide for ILF NF (Othmer, 2017) with individual adjustment of the exact training frequency based on the clinical response in terms of reported over- or under-arousal symptoms. Following the protocol guide, electrode placement at T4-P4 was applied, which is generally recommended for trauma patients to promote physical calm, while placement at T3-T4 was applied to reduce instabilities of the nervous system, meaning paroxysmal symptoms such as dissociation or panic attacks, which were reported prior to treatment by all participants. Placement at T4-Fp2 was used tentatively in order to achieve emotional calm and was only continued if the participants found it helpful.

**Figure 1:**
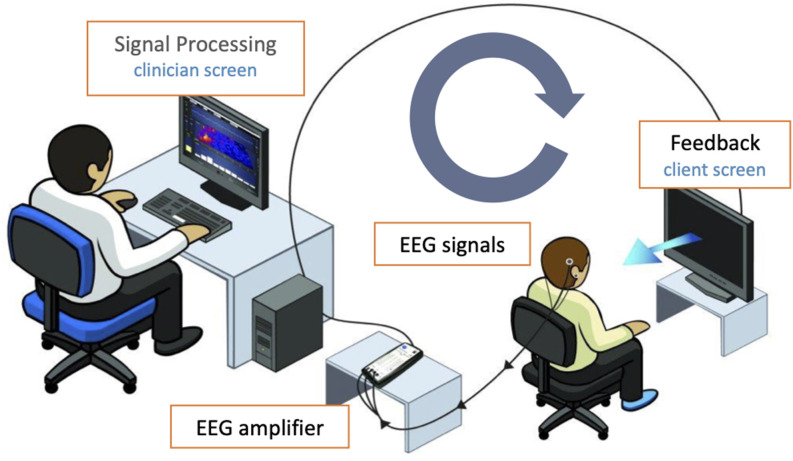
Neurofeedback setup (image courtesy of BEE Medic GmbH).

In the NF session, participants received EEG feedback watching either the InnerTubeNF game or the Particle Shapes NF animation (Somatic Vision Inc., Encinitas, CA, USA) according to personal preference. In InnerTube, the participant watches a rocket moving through tunnels, with the rocket’s speed being determined in real-time by the infra-low frequency band-limited waveform of the EEG signal. In Particle Shapes, nature scenes or abstract animations become more colorful and larger or paler and smaller, in the same way, depending on the EEG signal. Since ILF NF is based on implicit learning processes, there was no instruction to intentionally manipulate the parameters of the feedback. Participants were told that the changes in feedback should only be taken as information about their brain activity and not as an indicator of the quality of the session or of their performance (see Figure 1 for the neurofeedback setup).

#### “Media-Supported Relaxation”

A placebo intervention named “Media-Supported Relaxation” (MSR) was designed for this study to make external conditions as similar as possible to the ILF NF. Each participant could choose one of a selection of five calm video depictions (scenes of nature such as forest, meadow, coral reef, mountains, and open fire), most of which were similar to the animations of the ILF NF condition, which was then presented to her. Additionally, it was possible to choose between musical accompaniment and nature sounds. The instruction was to sit in a relaxed position and watch the video. The participants were able to select a different video or to end a session early at any time if they did not find it pleasant. After 30 min, the video was stopped and the patient was asked to what extent her condition had changed during the session.

#### Regular Inpatient Treatment Program

The regular inpatient treatment program, in which all participants of both groups participated, included individual psychotherapy (75 min per week) and group psychotherapy (150 min per week) as well as body awareness therapy, creative therapy, physical activity adapted to the physical condition, psychoeducation, nutritional counseling, and mealtime support as needed.

### Data Analysis

For data analysis, SPSS Version 24 was used. All tests were two-tailed and considered significant when *p* values were <0.05, with marginally significant *p* values (<0.10) also being mentioned. After checking the homogeneity of variances, 2 (*time*) × 2 (*group*) repeated measurement analyses of variance (rmANOVA) were conducted for each of the primary outcomes with *time* as a within-subjects factor (pre-treatment, post-treatment), *group* as a between-subjects factor (ILF NF, MSR), and *time* × *group* interaction. In the case of a significant interaction effect, *post-hoc* *t*-tests were calculated to determine the simple effects. As effect size for rmANOVAs, partial eta squared ($\eta_{p}^{2}$) was used, with $\eta_{p}^{2}$ ≥ 0.06 indicating a medium and $\eta_{p}^{2}$ ≥ 0.14 indicating a large effect. To investigate the secondary outcomes, *t*-tests and Fisher’s exact test were used. The effect sizes *d* and φ were given, respectively, with |d| = 0.50 and |φ| = 0.30 indicating a medium effect and |d| = 0.80 and |φ| = 0.50 indicating a large effect (Cohen, 1988).

## Results

### Participant Flow

Patients newly admitted to inpatient treatment were successively approached for the study until the planned sample size of *N* = 36 patients had been reached. *N* = 39 patients were addressed in this way. *N* = 38 agreed to participate. These were (also successively) assigned equally to the ILF NF group and the MSR group. Two patients (one from each group) were excluded as the provisional diagnosis of PTSD could not be confirmed. Thus, data of the remaining *N* = 36 participants were included in the analysis. One patient in the MSR group did not complete the EDE-Q post-measurement. Therefore, the sample was reduced to *N* = 35 when analyzing the subscales of the EDE-Q. Because the completion of the discharge questionnaire was voluntary, only data from *N* = 14 participants were available for the GAPS-scale (*n* = 9 from the ILF NF-group; *n* = 5 from the MSR-group).

**Figure 2:**
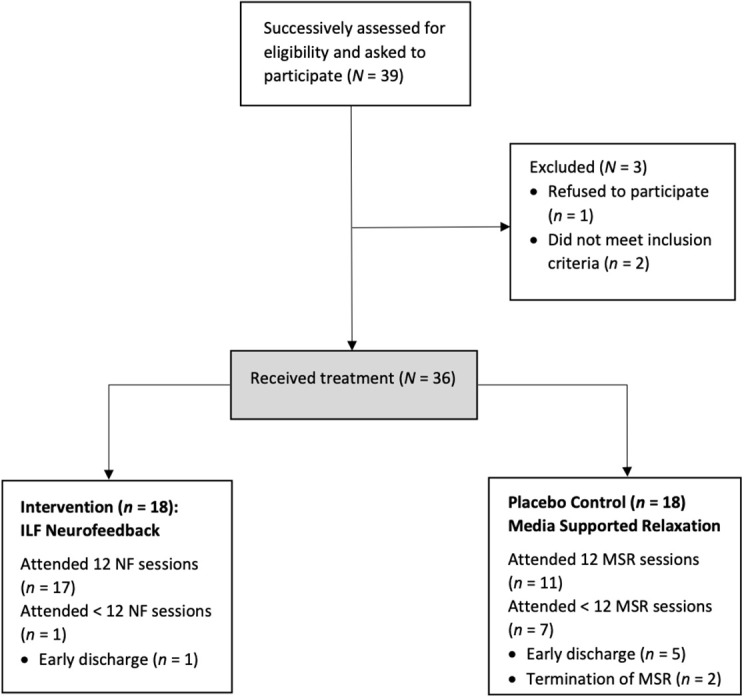
Participant flow.

Of the total *N* = 36 patients, *n* = 28 completed all 12 sessions of the planned intervention. Of the *n* = 18 patients in the ILF NF group, *n* = 17 completed all sessions of the ILF NF, *n* = 1 patient was discharged from inpatient treatment before completing the full number of sessions. Of the *n* = 18 patients in the MSR group, *n* = 11 completed all scheduled sessions, *n* = 5 patients were discharged from inpatient treatment before the full number of sessions was reached, *n* = 2 patients stopped taking part in the MSR at their own request, but remained in inpatient treatment. See Figure 2 for a presentation of the participant flow.

### Baseline Data

The patients whose data were included in the analysis (*N* = 36) had a mean age of 28.36 years (std.dev. 5.89). On average, they had been suffering from an ED for 13.19 years (std.dev. 7.41), had attended 6.9 (std.dev. 5.4) inpatient pre-treatments, and had experienced their first traumatic event at a mean age of 8.14 years (std.dev. 6.24). See Table 1 for sample characteristics. There were no significant differences between the two groups for these characteristics. However, the groups differed significantly in terms of BMI at the beginning of the treatment; the mean BMI was 18.22 kg/m^2^ (std.dev. 3.81) in the ILF NF group and 22.59 kg/m^2^ (std.dev. 8.04) in the MSR group, *t*_(24.26)_ = −2.08 (*p* < 0.05; a Welch-corrected *t*-test was used due to heteroskedasticity). Because no distinction had been made according to the type of ED in the randomized assignment to the groups, underweight, normal weight, and overweight patients were coincidentally distributed differently within the two groups.

**Table 1:** Illness-related characteristics of the sample.

**Variable**	**ILF NF**		**MSR**		**Total**	
	**Mean**	**Std. Dev**.	**Mean**	**Std. Dev**.	**Mean**	**Std. Dev**.
Age	27.11	5.28	29.61	6.34	28.36	5.89
Years of illness—eating disorder	11.67	6.31	14.72	8.27	13.19	7.41
Number of inpatient pre-treatments	6.61	3.29	7.11	6.95	6.86	5.37
Year of life—first trauma	8.28	5.96	8.00	6.69	8.14	6.24
BMI at admission	18.22	3.81	22.59	8.04	20.41	6.58

From the total sample, *n* = 17 participants were underweight at the onset of their inpatient treatment with BMI <18.5 kg/m^2^, thereof *n* = 10 belonged to the ILF NF group and *n* = 7 belonged to the MSR group. In this reduced sample, there was no significant difference in the BMI values between the two groups at the first measurement point, *t*_(15)_ = −0.50 (*p* > 0.60), and the Levene-Test indicated that variances in both groups were homogeneous (*p* > 0.80).

All *N* = 36 participants had a diagnosed ED, *n* = 33 had full PTSD, *n* = 3 had incomplete PTSD. All diagnoses were made according to ICD-10. The distribution of the various diagnoses between the two groups, including additional comorbidities (most commonly depression and emotionally unstable personality disorder), is shown in Table 2. On average, the participants fulfilled the diagnostic criteria for three mental disorders according to ICD-10. With regard to the type of traumatization experienced, *n* = 11 (31%) of the patients had been victims of sexual assault within their family of origin, *n* = 20 (56%) had been victims of sexual assault outside of their family, *n* = 18 (50%) had experienced non-sexual physical violence, *n* = 15 (42%) had experienced psychological violence, *n* = 6 (17%) had been neglected in their childhood, *n* = 1 (3%) had witnessed violence and *n* = 5 (14%) had experienced other traumatic events (multiple choices possible). All participants had suffered severe traumatic experiences that had lasted for a long period of time (so-called type II traumata) and met the criteria for complex PTSD according to ICD-11 (Maercker et al., 2013).

**Table 2:** Mental and behavioral disorders in the sample.

**Disorder**	**ICD-10 code**	**ILF NF**	**MSR**	**Total**
	*f*	*f*	*f*
**Eating disorders**
Anorexia nervosa	F50.0	9	6	15
Atypical anorexia nervosa	F50.1	3	3	6
Bulimia nervosa	F50.2	5	8	13
Atypical bulimia nervosa	F50.3	1	0	1
Other eating disorders	F50.8	0	1	1
Total		18	18	36
**Reactions to severe stress**
Post-traumatic stress disorder (PTSD)	F43.1	15	18	33
Other reactions to severe stress (incomplete PTSD)	F43.8	3	0	3
Total		18	18	36
**Other disorders**
Emotionally unstable personality disorder	F60.3	7	5	12
Recurrent depressive disorder, moderate	F33.1	11	11	22
Recurrent depressive disorder, severe without psychotic symptoms	F33.2	3	0	3
Social phobias	F40.1	3	1	4
Obsessive-compulsive disorder: Predominantly compulsive acts	F42.1	2	0	2
Persistent somatoform pain disorder	F45.4	0	1	1
Total		26	18	44

*f: frequency with which each disorder was diagnosed in the respective (sub)sample(s)*.

All dependent variables were examined for deviations from the normal distribution and for any outliers. There were no relevant abnormalities here. In addition, the intercorrelations of the questionnaires’ subscales were calculated. All EDE-Q-subscales showed substantial intercorrelations from *r* = 0.50 to *r* = 0.83 (*p* ≤ 0.001), with the subscale *weight concern* sharing the highest correlation coefficients with the other subscales. Thus, *weight concern* was excluded from the further analysis in order to avoid redundancy in the results. After that, the highest of the remaining intercorrelations was *r* = 0.57 which was tolerable. At the IES-R only the subscales *hyperarousal* and *intrusion* were significantly correlated (*r* = 0.53; *p* = 0.001), whereas *avoidance* showed no significant intercorrelation with *hyperarousal* (*r* = −0.06; *p* > 0.70) and *intrusion* (*r* = 0.14; *p* > 0.40).

### Primary Outcomes

#### Eating Disorder Psychopathology

**Figure 3:**
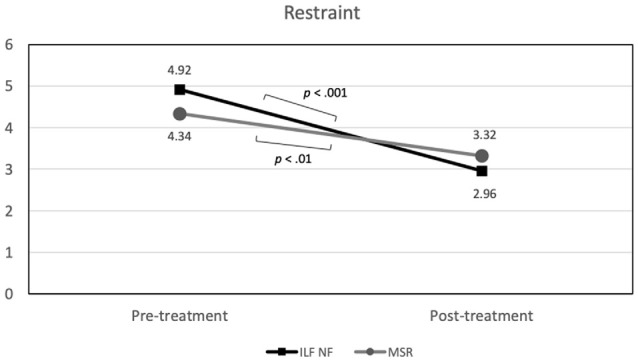
Group × Time interaction effect on restraint (significant mean differences are indicated).

In terms of *restraint*, a very large effect of *time* emerged, *F*_(1,33)_ = 56.07 (*p* < 0.001; $\eta_{p}^{2}$ = 0.63), accompanied by a significant *time* × *group* interaction, *F*_(1,33)_ = 5.58 (*p* < 0.05; $\eta_{p}^{2}$ = 0.15), whereas a main effect of *group* was not visible (see Table 3 for an overview of all effects on primary outcomes). As illustrated in Figure 3, both groups showed a notable reduction of restrained eating behavior between beginning and end of the planned treatment sessions. Accordingly, individual comparisons between the two measurement time points using *t*-tests for dependent samples became significant for both the NF group, *t*_(17)_ = 6.72 (*p* < 0.001) and the MSR group, *t*_(17)_ = 3.79 (*p* < 0.01).Though, this reduction was somewhat stronger in the ILF NF group than in the MSR group, i.e., for ILF NF mean was 4.92 (std.dev. 1.17) for pre-treatment and 2.96 (std.dev. 1.00) for post-treatment, whereas in the MSR group mean was 4.34 (std.dev. 1.50) for pre-treatment and 3.32 (std.dev. 1.26) for post-treatment. However, there were no differences between the groups at the individual measurement times; neither at pre-treatment, *t*_(34)_ = 1.18 (*p* > 0.80) nor at post-treatment, *t*_(33)_ = −0.95 (*p* > 0.30).

**Table 3:** Repeated measures analyses of variance on primary outcomes.

**Dependent variable**	**Time**		**Group**		**Time × Group**	
**Eating disorder psychopathology (*N* = 35)**
	**F_(1,33)_**	** $\eta_{p}^{2}$ **	**F**_(1,33)_	** $\eta_{p}^{2}$ **	**F**_(1,33)_	** $\eta_{p}^{2}$ **
Restraint	56.07***	0.63	0.09	0.00	5.58*	0.15
Eating concern	26.74***	0.45	0.65	0.00	0.89	0.03
Shape concern	5.57*	0.14	0.51	0.02	0.03	0.00
**Post-traumatic stress symptoms (*N* = 36)**
	**F_(1,34)_**	** $\eta_{p}^{2}$ **	**F**_(1,34)_	** $\eta_{p}^{2}$ **	**F**_(1,34)_	** $\eta_{p}^{2}$ **
Intrusion	0.05	0.00	0.20	0.01	0.00	0.00
Avoidance	3.23^+^	0.09	1.92	0.05	3.91^+^	0.10
Hyperarousal	4.61*	0.12	0.98	0.03	0.70	0.02
**BMI change (*N* = 17)**
	**F_(1,15)_**	** $\eta_{p}^{2}$ **	**F**_(1,15)_	** $\eta_{p}^{2}$ **	**F**_(1,15)_	** $\eta_{p}^{2}$ **
BMI	13.35**	0.47	1.17	0.01	3.82^+^	0.20

**F**: *F*-value for the respective effect with specification of the numerator and denominator degrees of freedom. **$\eta_{p}^{2}$**: partial eta squared. ***p < 0.001; **p < 0.01; *p < 0.05; ^+^*p* < 0.10.

Relatively large effects of *time* were determined also for *eating concern*, *F*_(1,33)_ = 26.74 (*p* < 0.001; $\eta_{p}^{2}$ = 0.45) and somewhat smaller for *shape concern*, *F*_(1,33)_ = 5.75 (*p* < 0.05; $\eta_{p}^{2}$ = 0.14). The frequency of eating-related cognitions was reduced substantially from pre-treatment to post-treatment, respectively. In particular, eating concern had a mean of 4.43 (std.dev. 1.10) at pre-treatment and a mean of 3.54 (std.dev. 1.24) at post-treatment, shape concern had a mean of 5.32 (std.dev. 0.90) at pre-treatment and a mean of 5.03 (std.dev. 0.90) at post-treatment. No significant effects of *group* and no significant *time* × *group* interactions were found for these variables.

#### Post-Traumatic Stress Symptoms

For *hyperarousal*, a significant effect of *time* was visible, *F*_(1,34)_ = 4.61 (*p* < 0.05; $\eta_{p}^{2}$ = 0.12). Hyperarousal after the 12 intended treatment sessions (post-treatment) was somewhat lower (mean 25.78; std.dev. 5.82) than at pre-treatment (mean 27.78; std.dev. 3.79). There was neither a significant effect between the two treatment groups nor a significant *time* × *group* interaction.

**Figure 4:**
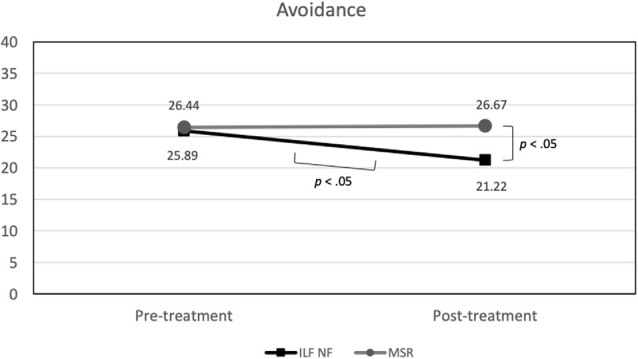
Group × Time interaction effect on avoidance (significant mean differences are indicated).

With regard to *avoidance* there was also no significant effect of *group*. However, a marginal significant effect of *time*, *F*_(1,34)_ = 3.23 (*p* = 0.08; $\eta_{p}^{2}$ = 0.09) emerged, which was qualified by a substantial but likewise only marginally significant *time* × *group* interaction, *F*_(1,34)_ = 3.91 (*p* = 0.06; $\eta_{p}^{2}$ = 0.10). As can be seen in Figure 4, the ILF NF group showed a significantly lower level of avoidance at post-treatment (mean 21.22; std.dev. 8.81) compared to pre-treatment (mean 25.89; std.dev. 7.64), *t*_(17)_ = 2.69 (*p* < 0.05) while the level of avoidance in the MSR group was virtually not different between the two measurement points, pre-treatment mean 26.44 (std.dev. 6.72), post-treatment mean 26.67 (std.dev. 6.54), *t*_(17)_ = −0.13 (*p* > 0.90). Both groups did not differ significantly in their avoidance at beginning of treatment, *t*_(34)_ = −0.23 (*p* > 0.80), whereas at the end of the treatment the ILF NF group was significantly lower in avoidance than the MSR group, *t*_(34)_ = −2.11 (*p* < 0.05).

#### Body Mass Index

**Figure 5:**
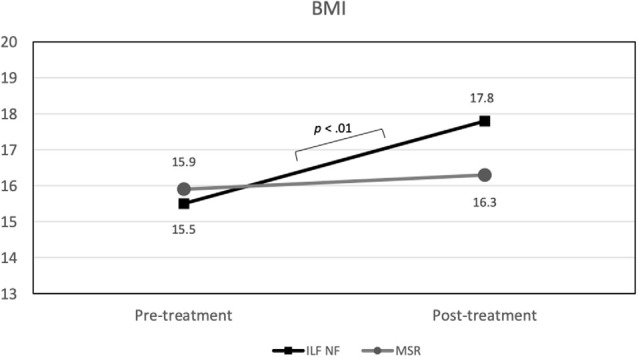
Group × Time interaction effect on BMI (significant mean differences are indicated).

In the rmANOVA, a substantial effect of time on BMI emerged, *F*_(1,15)_ = 13.35 (*p* < 0.01; $\eta_{p}^{2}$ = 0.47) as well as a marginally significant effect of the *time* × *group* interaction, *F*_(1,33)_ = 3.82 (*p* = 0.07; $\eta_{p}^{2}$ = 0.20). Again, the main effect of *group* was not significant. As Figure 5 shows, the ILF NF group achieved a significant increase in BMI from pre-treatment (mean 15.50; std.dev. 1.78) to post-treatment (mean 17.81; std.dev. 2.31), *t*_(9)_ = −3.95 (*p* < 0.01). In contrast, the BMI values in the MSR group did not differ significantly between pre-treatment (mean 15.93; std.dev. 1.71) and post-treatment (mean 16.30; std.dev. 2.22), *t*_(6)_ = −1.36 (*p* > 0.20). Since the variances were homogeneous and the BMI values were approximately normally distributed in both groups, the *t*-tests could be performed despite the small sample size (de Winter, 2013). The difference in the BMI values between both groups at the end of the intended treatment was not significant, *t*_(15)_ = 1.05 (*p* > 0.30).

### Secondary Outcomes

#### Perceived Benefit From Intervention

Participants in the ILF NF group reported a mean benefit from the NF sessions of 7.22 (std.dev. 1.87) that was significantly higher than the benefit reported by participants in the MSR group with regard to the placebo intervention (mean 3.83; std.dev. 2.53), *t*_(34)_ = 4.58 (*p* < 0.001; *d* = 1.52).

#### Patients’ Global Assessment of Psychological Treatment Success

At the end of their inpatient stay, the participants in the ILF NF group showed lower values on the GAPS scale (mean 2.00; std.dev. 0.54) than the participants in the MSR group (mean 2.68; std.dev. 0.88). In other words, patients who had received ILF NF rated their global psychological treatment success somewhat more favorably and perceived themselves on the average as "slightly improved" with regard to the aspects of mental condition assessed in the GAPS, while the patients in the MSR group perceived themselves as nearly unchanged. In the reduced sample this difference was marginally significant, *t*_(12)_ = −1.82 (*p* = 0.09; *d* = 1.01).

#### Complications in the Course of Treatment

Complications occurred in *n* = 5 participants in the MSR group (non-compliance with weight agreement in four cases, severe self-injurious behavior in one case) but none in the NF group. A Fisher’s exact test was performed which showed a significant relationship between treatment condition and occurrence of complications (*p* = 0.045, *φ* = 0.40).

## Discussion

This study is one of the few to systematically examine the incremental effects of implementing NF in a multimodal inpatient treatment setting, similar to that of Lackner et al. (2016), but using a placebo control condition rather than a treatment as usual control condition. Our results show that patients with ED and co-occurring PTSD treated with 12 sessions of ILF NF improved significantly better in restrained eating behavior than the patients treated with a placebo intervention. For patients who were underweight before treatment, the results also show that those in the ILF NF condition tended to gain more weight than those in the placebo condition. Regarding the effect of NF on restrained eating, our findings are at first glance consistent with those of Lackner et al. (2016) who found a significant effect of alpha frequency training on the restriction and dieting scale of the Eating Disorder Cognition Questionnaire. However, the restraint scale we used focuses more on behavior compared to the scale used by Lackner et al. (2016). Given that the tendencies concerning BMI development could only be found in our study, it could be assumed that the ILF method has a stronger effect on actual eating behavior and thus also on weight gain than alpha frequency training.

Furthermore, we found that the extent of avoidance as one of the core components of PTSD symptoms tended to be reduced more in the ILF NF condition than in the MSR condition, leading to a significantly lower avoidance in the ILF NF condition post-treatment compared to the MSR condition. Concerning hyperarousal and intrusions according to IES-R, no superiority of the ILF NF condition could be shown, which contradicted our expectations with regard to the strong effects of NF on the general PTSD symptoms in chronic PTSD reported by van der Kolk et al. (2016). However, it should be taken into account that in the study of these authors, twice as many NF sessions were carried out with each participant than in the present one. A higher number of sessions may have to be conducted, especially in the case of a high degree of severity of the PTSD, as in our sample, in order to achieve a greater effect with regard to the basal level of arousal and the general excitability of the patient’s nervous system.

Previous studies found that neurofeedback resulted in increased calmness and physical relaxation (Kluetsch et al., 2014) and a decrease in tension reduction activities (van der Kolk et al., 2016), which is indirectly indicative of a decrease in hyperarousal. Thus, one could assume that this calming effect of NF would result in a decrease in feelings of fear and thereby lead to a reduction in trauma-associated avoidance. However, a decrease in avoidance in our ILF NF participants did not appear to be associated with a greater reduction in hyperarousal compared to the control condition. Therefore, it remains to be explained how the positive effect of ILF NF on trauma-associated avoidance is mediated. In view of the finding of Dobrushina et al. (2020), that ILF NF leads to increased connectivity of networks in the brain responsible for the processing and integration of sensory stimuli of different modalities, which are thereby presumably involved in the perception and recognition of emotions, ILF NF might lead to an increased awareness of both external stimuli and emotions, and thereby to a reduction in trauma-associated avoidance. There may as well be other effects of ILF NF which lead to a reduction in avoidance, e.g., in terms of a reduction in fear and negative expectations in the face of trauma-associated stimuli, or in the sense of a more positive assessment of one’s own abilities to face them. For the time being, the mechanisms of action remain speculative and must be elucidated by future more specific surveys of possible mediating variables, including other studies using imaging techniques such as fMRI.

In addition to revealing some encouraging effects of ILF NF compared to the placebo control group, this study clearly demonstrates for the entire sample that a multimodal inpatient treatment program can have major effects in terms of reducing the burden of core symptoms of the disorders examined here. This is primarily reflected in restrained eating behavior. It can be assumed that the behavior-controlling effect of the treatment setting had the greatest impact here since regular meals under supervision are the most likely to bring about a change. But even with symptoms of ED that respond more to psychotherapeutic treatment, significant improvements can be observed, such as eating concern or shape concern. Moreover, we found that hyperarousal significantly decreased from the beginning to the end of treatment for all participants so that the assumption that the lowering of hyperarousal is a decisive prerequisite to achieve further therapy effects on trauma-related distress and eating behavior can be further maintained, yet there was no additional effect for patients receiving ILF NF.

One of the strengths of our study is that it was embedded in an existing treatment setting and examined an unselected population of newly admitted inpatients fulfilling the ICD-criteria of both ED and PTSD so a high external validity has to be assumed. In contrast to several comparable studies, the ILF NF condition was subjected to a rigorous test here, being not only compared to treatment as usual, but to a placebo condition in order to rule out the possibility that any effect of ILF NF was solely due to an associated increase in therapy units for the participants.

With regard to the placebo condition, it should be noted that MSR showed a strong similarity to the ILF NF condition in many features (comfortable armchair in front of a monitor, visual and acoustic impressions), but was recognizable to the participants as different from it, in particular, due to the lack of electrodes. Therefore, a control group using sham ILF NF (in which the patients are only in appearance connected to the NF apparatus and receive visual and acoustic “feedback”, which is in fact randomly generated) would have been even more suitable for examining the specific effects of ILF NF.

The study was based on test scores of well-established self-report questionnaires that are part of the routinely used test battery of the clinic. However, for the EDE-Q, an examination of the intercorrelations of the subscales revealed that the three dimensions that revolve around concerns seem to have not only high conceptual but also substantial empirical overlaps so we decided to exclude the weight concern scale from the further analysis. For future studies, a supplementation of the assessment of eating disorder psychopathology with other instruments would be desirable.

The most significant limitation of the study is probably the lack of homogeneity within the examined sample with regard to the ED diagnoses and the associated BMI distribution between the groups. To analyze the change in BMI, the already small sample had to be further reduced in size, which limited the power. From this point of view, further studies should be carried out with samples that are more homogeneous in terms of BMI, which, however, in our treatment setting would have lengthened the total duration of data collection significantly due to the exclusion of potential participants with normal or high body weight.

In conclusion, it can be stated that with regard to the central aim of the study, indications of incremental treatment effects of ILF NF on ED psychopathology and trauma-related stress in an inpatient setting could be found. In addition, it could be shown that this type of implicit NF was very well accepted by the patients and was associated with a significantly lower rate of severe complications and, in tendency, a more positive assessment of the global psychological treatment outcome. Patients who received ILF NF seemed more inclined to classify themselves as “slightly improved” at the end of their inpatient treatment, indicating that they had experienced a clinically meaningful change (cf. Haase et al., 2021). These preliminary results show the basic feasibility of the applied research design and encourage further studies with larger and more homogeneous samples and a larger number of NF sessions with extended observation time. Beyond the more reliable proof of effectiveness, the mechanisms of action of this promising therapeutic approach still require further elucidation.

## Data Availability Statement

The raw data supporting the conclusions of this article will be made available by the authors, without undue reservation.

## Ethics Statement

The studies involving human participants were reviewed and approved by Ethical Review Board of the Landesärztekammer Hessen (regional medical association); Reference number FF 121/2018. The patients/participants provided their written informed consent to participate in this study.

## Author Contributions

AW conceived the idea, planned the study design, recruited the participants, collected the data, and drafted the manuscript. MW conducted the data analysis and contributed to the writing of the manuscript. HI critically reviewed the manuscript and supervised all procedures. All authors revised and approved the final manuscript. All authors contributed to the article and approved the submitted version.

## Conflict of Interest

The authors declare that the research was conducted in the absence of any commercial or financial relationships that could be construed as a potential conflict of interest.

## Publisher’s Note

All claims expressed in this article are solely those of the authors and do not necessarily represent those of their affiliated organizations, or those of the publisher, the editors and the reviewers. Any product that may be evaluated in this article, or claim that may be made by its manufacturer, is not guaranteed or endorsed by the publisher.

## Footnotes

^1^ https://www.random.org
